# Sensitivity of the fasciae to sex hormone levels: Modulation of collagen-I, collagen-III and fibrillin production

**DOI:** 10.1371/journal.pone.0223195

**Published:** 2019-09-26

**Authors:** Caterina Fede, Carmelo Pirri, Chenglei Fan, Giovanna Albertin, Andrea Porzionato, Veronica Macchi, Raffaele De Caro, Carla Stecco

**Affiliations:** 1 Department of Neurosciences, Institute of Human Anatomy, University of Padova, Padova, Italy; 2 Physical and Rehabilitation Medicine, University of Rome “Tor Vergata”, Roma, Italy; Georgia State University, UNITED STATES

## Abstract

Although it is now recognized that women suffer from myofascial pain to a greater extent than men, and that the muscular fasciae can respond to hormonal stimuli, thanks to the expression of sex hormone receptors, how the fasciae can modify their structure under hormonal stimulation is not clear. In this work, an immunocytochemical analysis of collagen-I, collagen-III and fibrillin were carried out on fibroblasts isolated from human fascia lata after *in vitro* treatment with various levels of sex hormones β-estradiol and/or relaxin-1, according to the phases of a woman’s period (follicular, periovulatory, luteal, post-menopausal phases and pregnancy). This study demonstrates for the first time that fascial cells can modulate the production of some components of the extracellular matrix according to hormone levels, when treated with β-estradiol: collagen-I falls from 6% of positivity in the follicular phase to 1.9 in the periovulatory phase. However, after the addition of relaxin-1 to the cell culture, the production of extracellular matrix decreased and remained at the same level (1.7% of collagen-I, at both follicular and periovulatory levels of hormones). These results confirm the antifibrotic function of relaxin-1, thanks to its ability to reduce matrix synthesis. They are also a first step in our understanding of how some hormonal dysfunctions in women can cause a dysregulation of extracellular matrix production in fasciae.

## Introduction

Estrogens have long been known as a regulating factor of metabolism in tissues such as bone, muscle, cartilage, tendon and ligament, affecting the musculoskeletal functions [1]. The estrogen-beta receptor has also been described recently in the tissue of tendons and ligaments [2], and only in one of our recent works [3] the expression of sex hormone receptors was also demonstrated in the muscular fasciae. It was recently observed that knee laxity is significantly greater around ovulation, when estrogen peaks [4], with an increased risk of injury [5]. Konopka et al. [6] demonstrated that female collegiate athletes with serum relaxin concentrations above 6.0 pg/mL are more than four times as likely to suffer from anterior cruciate ligament (ACL) tears. The rapid changes of laxity during the cycle may be due to any significant change in collagen content within days. Another study has shown that short-term exposure to physiological concentrations of estrogen decreases the mechanical function of ligaments by inhibiting the activity of the crosslinking enzyme lysyl oxidase, which contributes to the increased laxity seen in the female knee [7].

Several studies have evaluated the effect of female sex hormone supplementation in tendons: some authors report that estradiol has an inhibitory effect on collagen synthesis and fibroblasts proliferation in ACL tissue [8,9]. Instead, Lee et al. [10] have shown that cyclic tensile loading applied to fibroblasts increases the mRNA expression of collagen-I, but not that of collagen-III or biglycan: estrogen increased the mRNA expression of collagen-I and III and did not change that of biglycan, but the combined effects of cyclic tension and estrogen inhibited the mRNA expression of these three molecules.

Another work [11] demonstrated the variability of daily myofascial pain, which was more constant in oral contraceptive users with respect to non-users: the latter have peaks of pain alternating with pain-free periods within the menstrual cycle, with many variations in pain, whereas patients who used oral contraceptives were significantly more stable over time. Lee and Park [12] described how musculoskeletal pain characteristics are caused by the severity of menstrual pain. Lee and Petrofsky [13] demonstrated that changes in plantar fascia elasticity during the menstrual cycle may involve sexual hormones in the increasing elasticity of human connective tissue.

Various trails have shown that women treated with aromatase inhibitors, which stop the production of estrogen in post-menopausal women, often experience joint pain and musculoskeletal aching. These manifestations may be reduced after cessation of therapy, and one explanation for these findings is that a rapid drop in estrogen levels enhances nociception [14].

Evaluation of the effects of estrogen replacement therapy (ERT) in post-menopausal women demonstrates that it may influence tendon biomechanical properties and morphology, with smaller fibrils and higher fibril density, in contrast to the inhibiting effect of ERT on bone collagen synthesis. These observations indicate the possible role of estradiol in maintaining homeostasis in female connective tissue [15].

The aim of this work was to evaluate the effect of sex hormones, in particular beta-estradiol and relaxin-1, on the *in vitro* production of some elements of the extracellular matrix by human fascial fibroblasts, in relation to the hormone levels of the menstrual cycle. We aim at better understanding of how varying hormone levels can contribute to fascial tissue composition and consequently to fascial stiffness and sensitization of fascial nociceptors.

## Materials and methods

### Preparation of hormones for cell culture

According to indications (Sigma-Aldrich), hormone solutions were prepared as described below. Powder of β-estradiol was diluted in 1 ml absolute ethanol to prepare a solution of 1 mg β-estradiol, gently swirled to dissolve it and diluted in a 49-ml sterile medium during mixing, to obtain a 20-μg/ml stock solution. Relaxin-1 (lyophilized powder, recombinant, Sigma-Aldrich) was reconstituted in Phosphate Buffered Saline solution containing 0.1% serum albumin, to obtain a concentration of 10 μg/mL.

### Cell isolation and culture

Cells were isolated from human fascia lata of the thigh (~ 1cm × 1 cm) collected from a volunteer, female patient, aged 50 y, who was undergoing elective surgical procedure at the Orthopedic Clinic of University of Padova. The patient was in the premenopausal period, and was not on hormone replacement therapy. The ethical regulations regarding research conducted on human tissues were carefully followed (approval no. 3722/AO/16, study approved on 21 April 2016 by the Ethical Committee for clinical trials in the province of Padova), after written informed consent was obtained from the donor. The sample was transported to the laboratory in phosphate buffered saline (PBS) within a few hours of collection, and digested with Collagenase B 0.1% in Hank's Balanced Salt Solution (HBSS) overnight. Fibroblast cells were isolated and characterized by immunohistochemical staining with anti-Fibroblast Surface Protein[1B10] antibody (1:100, Mouse monoclonal antibodies, AbCam Cambridge, UK), as previously described in one of our previous works [16]. Cell cultures were maintained at 37°C, 95% humidity and 5% CO2, in DMEM 1g/L glucose, 10% FBS and 1% penicillin-streptomycin antibiotic, and used from passages 3 to 9.

### Cell treatment with sex hormones

Isolated cells from fascia were plated (200 cells/mm^2^ in 24-multiwells containing a glass coverslip) and allowed to attach for 48 h at 37°C. They were then maintained for 24 h in DMEM without serum, so as not to interfere with the treatment: endogenous hormone-binding proteins are present in varying concentrations in all serum and plasma samples and may markedly influence hormone treatments and assay results [17]. The cells were then treated for 48 h with β-estradiol and/or relaxin-1 human in DMEM without serum (see Table 1) [18–23]. In each experiment, one control sample underwent the same processing steps, but did not receive hormones. After treatment, cells were washed in PBS, fixed for 10 min with 2% paraformaldehyde in PBS, pH 7.4, and then washed three times in PBS before the staining protocols described below.

**Table 1:** Hormone levels. Average hormone levels during the women period, in pregnancy and after menopause.

	β-Estradiol (pg/mL)	Relaxin-1 (pg/mL)
**Post-menopausal period**	10	0
**Follicular phase**	90	20
**Periovulatory phase**	400	20
**Luteal phase**	150	150
**Pregnancy (2**^**nd**^ **trimester)**	5000 (range 4000–7000)	4000

### Collagen staining

Picrosirius red staining was first applied to visualize collagen: Picro-Sirius Red solution (0.1 g of Sirius Red- Sigma Aldrich per 100 mL of saturated aqueous solution of picric acid) was applied to fixed cells for 20 min, and then washed out with acidified water (5 mL acetic acid, glacial, to 1 liter of water).

### Immunocytochemistry

The immunocytochemistry procedure included the following steps: after blocking of endogen peroxidase by 0.5% H_2_O_2_ in PBS for 10 min at room temperature and repeated washes in PBS, samples were pre-incubated with a blocking buffer (0.1% bovine serum albumin, BSA, in PBS) for 60 min at room temperature. The cells were then incubated in: rabbit anti-collagen-I, N-terminal antibody (dilution 1:100, Sigma-Aldrich, Saint Louis, MO, USA), rabbit anti-collagen-III polyclonal antibody (dilution 1:100, Abcam, Cambridge, UK), or mouse anti-fibrillin monoclonal antibody (dilution 1:200, Merck Millipore, CA, USA). The primary antibodies were diluted in the same pre-incubation buffer and incubated overnight at 4°C. After repeated PBS washings, samples were incubated with the ready-to-use secondary antibody Advance HRP Detection System (Dako Corp., Carpinteria, CA, USA), and washed in PBS buffer. The reaction was then developed with 3,3’-diaminobenzidine (Liquid DAB + substrate Chromogen System kit Dako Corp.) and stopped with distilled water.

Negative controls were similarly treated, without the primary antibody, confirming the specificity of the immunostaining.

### Image analysis

In all samples nuclei were counterstained with ready-to-use hematoxylin (Dako Corp.). Images were acquired on a Leica DMR microscope (Leica Microsystems, Wetzlar, Germany; objectives 20X and 40X, Leica). Computerized image analysis was performed with ImageJ software to quantify anti-collagen-I, anti-collagen-III and anti-fibrillin antibody positivity (staining was repeated at least 3 times with each antibody, and for each sample at least 20 images were analysed, magnification 20X).

### Statistical analysis

Student’s *t* test was used to verify significant differences when comparing data of control cells (not treated with any hormone) and cells treated with varying levels of β-estradiol and/or relaxin-1.

## Results

**Fig 1:**
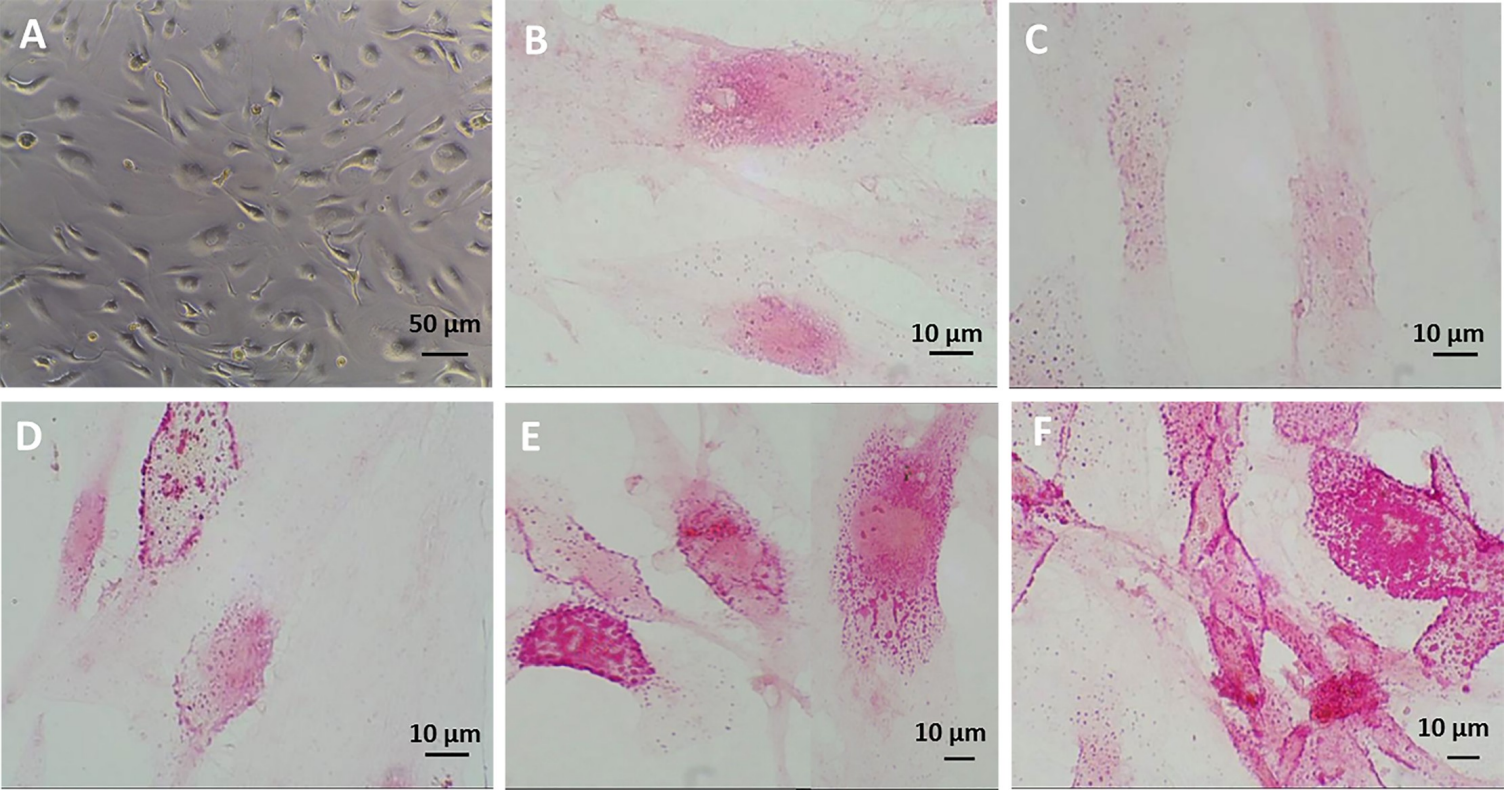
Picrosirius red staining after β-estradiol. Bright field image (A) of cell culture after hormone treatment. Picrosirius red staining (B, C, D, E, F) of fascial fibroblast after 48 h of treatment with β-estradiol: control cells (B), 10 pg/mL (C), 90 pg/mL (D), 400 pg/mL (E), 5000 pg/mL (F).

In Fig 1A is shown one bright field image of fascial fibroblasts seeded in multiwells and incubated with sex hormones in medium without serum: neither treatment nor the absence of serum affected cell growth in the *in vitro* culture, after cells attachment. The cells were not affected either by the absence of serum or hormone concentration: density and morphology remained the same with respect to cells maintained in culture with serum and without hormone incubation (data not shown).

Staining with Picrosirius red (Fig 1B, 1C, 1D, 1E and 1F) was used for histological visualization of collagen fibers synthesized by the cells, showing a cytoplasmic stains. The pictures revealed some modifications according to hormone levels: after treatment with low concentrations of β-estradiol (10 pg/mL, post-menopausal levels) (Fig 1C), the staining decreased with respect to control cells (Fig 1B), but was much more evident with increasing hormone levels up to 5000 pg/mL (Fig 1F), corresponding to the average amount of β-estradiol during the 2^nd^ trimester of pregnancy.

**Fig 2:**
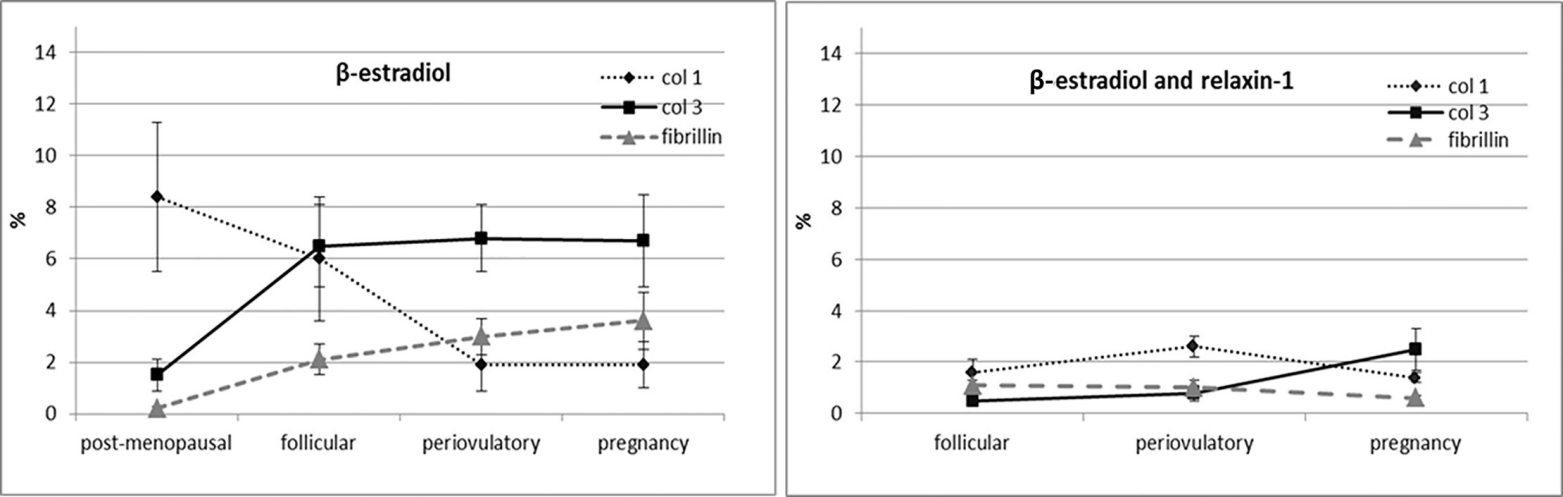
Curves of positivity to col I, col III and fibrillin: effect of relaxin-1. Trend of positivity to anti-collagen-I, anti-collagen-III and fibrillin antibody, according to hormonal phases of a woman, after treatment with β-estradiol only (left) or β-estradiol and relaxin 1 (right).

**Fig 3:**
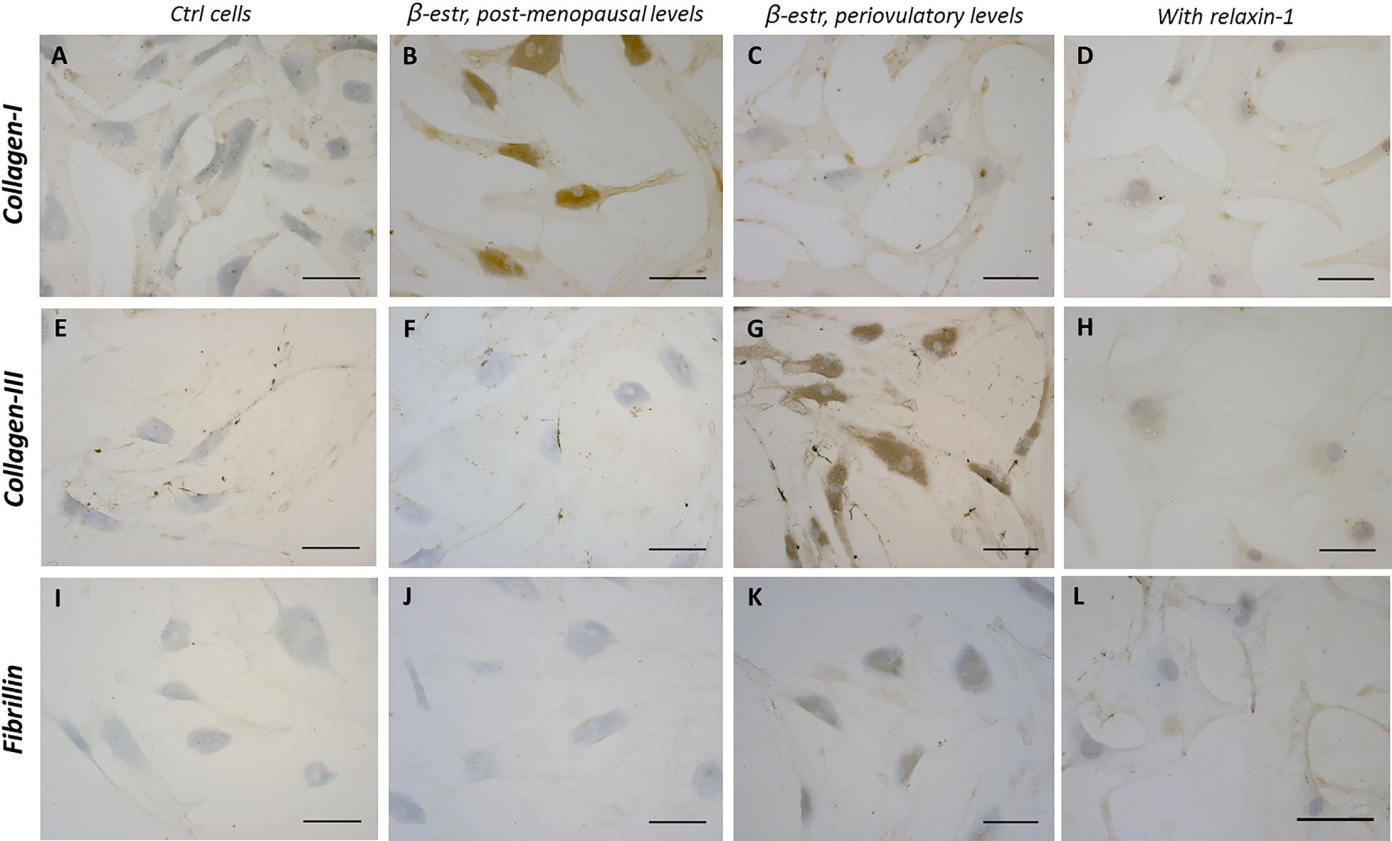
Immunostaining after hormone treatments. Collagen-I (A-D), collagen-III (E-H) and fibrillin (I-L) expression in fascial fibroblasts after 48 h of treatment with β-estradiol 10 pg/mL (B, F, J), β-estradiol 400 pg/mL (C, G, K), or β-estradiol 400 pg/mL+ relaxin-1 20 pg/mL (D, H, L). Control cells (A, E, I):cells not incubated with hormones. Scale bars: 100 μm.

According to these results, immunocytochemical analysis was applied to explore and quantify the amount of components of the extracellular matrix: collagen-I, collagen-III and fibrillin. All results are shown in Table 2 and in Figs 2 and 3. Fascial cells can modulate the production of the extracellular matrix: when treated for 48 h with β-estradiol, the amount of collagen-I, III and fibrillin changed greatly according to hormone levels. With a post-menopausal level of β-estradiol (~10 pg/mL) the amount of collagen-I rose to 8.4% of positivity (with a statistically significant variation from the starting percentage of 5.2 in control cells). Conversely the amount of collagen-III has significantly lowered compared with the control (1.5% of positivity, with respect to 2.4% of the control cells). Instead, with periovulatory or pregnancy hormone concentrations, the amount of different collagen types switched: collagen-III rose to 6.8 or 6.7% (periovulatory and pregnancy concentrations, respectively), whereas collagen-I fall to 1.9%. All these variations were statistically significant.

**Table 2:** Percentages of positivity to col I, col III and fibrillin according to hormone level. Percentage (%) of positivity to anti-col I, anti-col III, and anti-fibrillin antibodies of an *in vitro* cell culture of fascial fibroblasts, treated for 48h with various levels of β-estradiol (β estr), relaxin-1 (RX1), or β-estradiol and relaxin-1 (β estr + RX1). Control cells (ctrl) were incubated in cell culture medium without serum and without hormone treatment. Values are means and standard deviations of at least three independent experiments, analysed by ImageJ software. Underlined values are not significantly different from corresponding control value. All other values had a statistically significant difference: p<0.05, Student’s *t* test, treated *vs*. controls.

	Col-I (%)			Col-III (%)			Fibrillin (%)		
	β estr	RX1	β estr+ RX1	β estr	RX1	β estr+RX1	β estr	RX1	β estr+RX1
**Ctrl**	5.2±0.05			2.4±0.04			0.5±0.04		
**post-menopausal**	8.4±2.9	/	/	1.5±0.6	/	/	0.2±0.1	/	/
**follicular**	*6±2*.*4*	1.7±0.05	1.6±0.5	6.5±1.6	0.9±0.01	0.5±0.1	2.1±0.6	1.1±0.06	1±0.2.1
**periovulatory**	1.9±1.0	1.7±0.04	2.6±0.4	6.8±1.3	0.9±0.05	0.8±0.3	3±0.7	1.1±0.06	1±0.3
**pregnancy**	1.9±0.9	1±0.04	1.4±0.2	6.7±1.8	1.1±0.01	*2*.*5±0*.*8*	3.6±1.1	2.1±0.1	*0*.*6±0*.*1*

Fibrillin also increased with increasing β-estradiol levels (from 0.2 to 3.6%, in menopause and pregnancy, respectively).

When relaxin-1 was added to the cell culture, either alone or in combination with β-estradiol, the production of collagen-I, III and fibrillin decreased and remained at the same level in all the phases of the period. For example, with relaxin-1 stimulation only, the percentage of collagen-I fibers fell to 1.7% with hormone levels of the follicular phase, and remained at the same level even with periovulatory hormone levels.

In addition, when relaxin-1 was added to β-estradiol, the amount of collagen-III during the periovulatory phase became 0.8%, with respect to 6.8% with β-estradiol stimulation only. Fibrillin also fell from 3 to 1%, and collagen-I reached 2.6%.

In general, stimulation with relaxin-1 decreases the synthesis of all the collagen fiber components with respect to control cells, whereas the total amount of fibrillin remained similar to control levels (0.5%), reaching 1% of positivity with the hormone levels of the follicular and periovulatory phases.

## Discussion

This is the first study demonstrating that the human fascia can respond to various hormonal stimuli, regulating the production of some components of the extracellular matrix (ECM), collagen-I, collagen-III and fibrillin.

All matrix components (collagen fibers, proteoglycans/glycosaminoglycans, elastin, fibronectin, laminins, and several other glycoproteins) bind to each other in a complex and highly dynamic network which permits and regulates various cellular functions such as survival, growth, migration and differentiation [24]. The composition and structure of ECM determines tissue behavior, and ECM reorganization is essential for normal reproductive functions during pregnancy and parturition. Nallasamy and coauthors have demonstrated that progesterone and estrogen play distinct and complementary roles to regulate synthesis, processing, assembly and structural reorganization of both collagen and elastic fibers in the cervix during pregnancy, regulating the mechanical function of the tissue [25].

**Fig 4:**
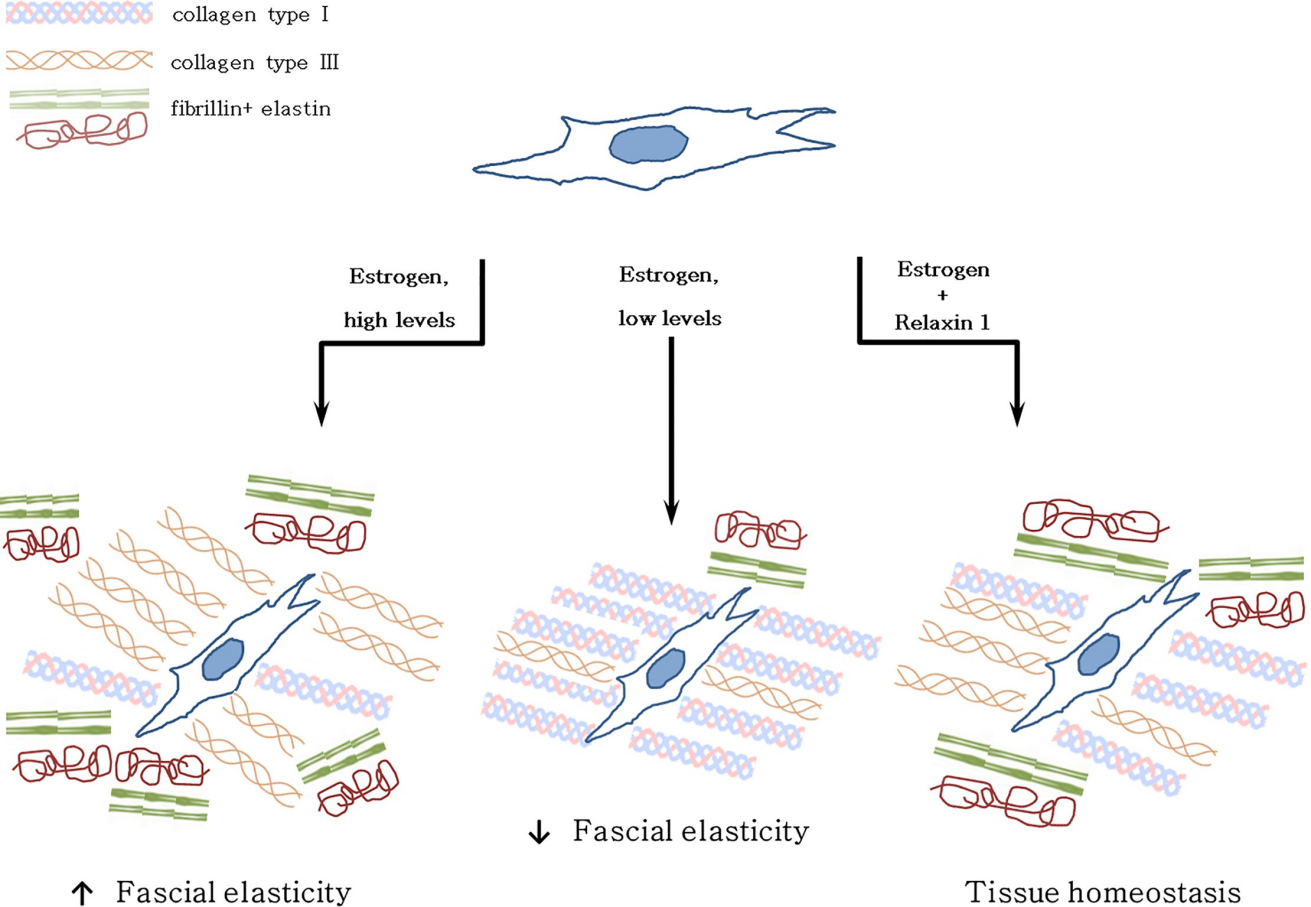
Fascia and ECM production according to hormone levels. Theoretical scheme of fascial cell response and extracellular matrix rearrangement at different hormone levels (estrogen and relaxin-1).

As we found that the fascia becomes enriched in collagen-I, with low hormone levels, the *in vivo* fascial tissue may probably become more rigid during menopause. Other studies related to the skin have already demonstrated that changes in collagen lead to diminished skin elasticity and strength [26], and remodeling of the collagenous network typical of tumor tissues is known to cause increased tissue stiffness [27]. If the connective tissue is altered, the behavior of the fascia and underlying muscle becomes compromised, being source of myofascial disorders in many cases [28]. These results are in line with other studies: a recent work demonstrated that vaginal fibroblasts isolated from postmenopausal women with pelvic organ prolapse show higher sensitivity and lower tolerance to mechanical stretching with respect to normal fibroblasts, with higher expression of collagen-I and a lower expression of collagen-III mRNA [29]. This work provides evidence supporting estrogen therapy to prevent and inhibit prolapse in postmenopausal women. ECM stiffening, induced by increased collagen deposition, especially collagen-I and cross-linking, disrupts normal tissue morphogenesis. In parallel, during menopause collagen-III and fibrillin contents decrease in a statistically significant way (from 2.4 to 1.5%, and from 0.5 to 0.2%, respectively), reducing the scaffolding which assists the alignment and cross-linking of elastin molecules [30], and consequently decreasing the elastic properties of fascial tissue (Fig 4).

Instead, when hormone levels rise, e.g. during pregnancy, the fascial tissue becomes more elastic: after administration of β-estradiol, collagen-I falls from 5.2 to 1.9%, whereas collagen-III and fibrillin increase (from 2.4 to 6.7%, and from 0.5 to 3.6%, respectively; see Fig 4). This change in ECM composition allows tissues to adapt during pregnancy process, like as during the ovulatory period, in which the same trend in ECM change can be observed: collagen-I falls from 5.2 to 1.9%, collagen-III increases from 2.4 to 6.8%, and fibrillin rises from 0.5 to 3%. A rigid fascia can help to stabilize the sacroiliac joint and the spine, as explained by Gracovetsky, who demonstrated how a rigid thoracolumbar fascia allows intracompartment pressure to increase: the extension force produced by expansion of the erector spinae muscles can thus contribute to the ability to lift a load [31,32]. So more lax fasciae may trigger pain at the pelvic or lumbar level, which is typical of pregnancy [33,34]. Another study by Marnach et al. [35] highlighted the development of joint pain during pregnancy in 35 women in the first-trimester: it was not associated with increased joint laxity, but with significantly increased levels of estradiol and progesterone. In the same way, fluctuations in estradiol were associated with aches, joint pain, stiffness, and depressed mood during the menopausal transition [36].

The surprising result which we obtained is that, if hormone relaxin-1 is added to the culture, these evident and rapid changes are reduced, as shown in Fig 4: from follicular hormone concentrations to pregnancy levels, there is a continuous decrease in collagen-I fibers and a corresponding increase in tissue elasticity, with collagen-III that goes from 0.5 to 2.5%. These slowed changes confirm the antifibrotic function of relaxin-1 by its ability to reduce matrix synthesis. Recent studies on relaxin-1 gene-knockout mice have, in fact, established this hormone as a naturally occurring moderator of collagen turnover, preventing fibrogenesis, but also improving organ structure and function, even in non-reproductive organs, including heart, lung, kidney, liver and skin [37]. It is possible that women with hormonal dysfunctions may present a dysregulation of extracellular matrix production, causing stiffness, fibrosis and inflammation which create sensitization of fascial nociceptors [38,39]. This may explain why oral administration of estrogen (the dose of β-estradiol via oral administration is usually 50 pg/mL) may resolve myofascial pain in women [40]. It has been demonstrated that there is a difference in ligamentous laxity between oral contraceptive pill (OCP) users and non-users: OCP users showed a statistically significant decrease in anterior translation of the tibia with respect to non-users, demonstrating the role of OCP in the prevention of anterior cruciate ligament injuries [41]. This is in line with our results, which confirm that hormonal imbalance damages myofascial tissue, leading to drastic changes in its constitution in collagen and elastic fibers, and thus modifying its biomechanical properties. Other authors have confirmed that the balance of estrogen α/estrogen β affects the physiological action of estradiol in all estrogen-responsive tissues [42].

Our findings are limited because this is an *in vitro* study, in cells derived from only one patient. Although in literature it is demonstrated that the fibroblasts replication in culture does not correlate with donor age [43], we expect differences in responsiveness depending on the age and period of the patients as we have demonstrated in our previous work, in which the expression of the receptors for estrogen and for relaxin-1 resulted lower in post-menopausal women, according to the decrease in estrogen hormone levels [3]. Although in the next future this work will be deepened by repeating the analyzes on other cells isolated from premenopausal women and then comparing the results with samples of post-menopausal women, we have chosen for this pilot *in vitro* study the cells that in our previous work had shown a homogeneous and high positivity of expression for hormone receptors [3]. Furthermore we analysed only two hormones (β-estradiol and relaxin-1), with no reference to the extracellular assembly and to the degradation of the collagen fibers, essential to determine the organization of the ECM and consequently the stiffness of a tissue [44]. Study of the complex multifactorial effect of sex hormones will clarify the association of sex hormones with other hormonal factors, with other elements of the ECM, or with patients age.

Hansen and co-authors have demonstrated that the administration of oral contraceptives (OC) to young women was associated with lower tendon collagen synthesis, probably because the use of OC is associated with lower insulin-like growth factor-1 (IGF-I), which enhances tendon collagen synthesis [45]. Ozturk et al. analysed the effect of Tamoxifen, a synthetic non-steroidal antiestrogen used primarily in the treatment of breast cancer, on fibrotic disease via downregulation of TGF1β, with inhibition of fibroblast proliferation and lower collagen density [46]. Other authors have analysed the dependence of the effects on women’s age: estrogen reduces tendon and ligament stiffness, so young women probably have a greater risk of ACL rupture. Instead, in post-menopausal women, estrogen/hormonal replacement therapy may counteract degenerative changes in skeletal muscle, with beneficial effects on skeletal muscle protein maintenance, muscle mass and strength, in preventing tendinopathy, and reducing tendon stiffness [47].

## Conclusions

Our further studies will hopefully shed light on the multifactorial mechanism of sex hormones and how they affect the fascia. However, the present work may help to explain some links between hormonal factors and remodeling of fascial matrix, leading in the next future to novel pharmaceutical and mechanical approaches for treating myofascial disorders, which consider how the diversity of hormone levels can affect the properties of the fascial tissue.
